# Erie County Medical Society

**Published:** 1873-01

**Authors:** 


### Editorial.

### Erie County Medical Society.

This Society held its Annual Meeting January 14th, the President, Dr. WILLIAM RING, presiding. Minutes of the last meeting were read by the Secretary, Dr. DAVID CHACE.

The following Physicians were admitted members upon compliance with the By-Laws: R. F. Erdmann, of Buffalo; Horace Grant Hopkins, and D. C. Hunter, of Clarence.

Report of Treasurer was read and approved. Librarian's Report was also read and approved. The Librarian recommended distribution to intelligent citizens, of the extra copies of Transactions of 1870.

Report of committee on admission of Dr. Harris as member of the Society, was read and approved. The following is the report of the Committee, and as it includes legal opinion, we publish the report as presented.

BUFFALO, January 14th, 1873.

*To the Erie County Medical Society:*

At the last semi-aunual meeting of the Society, the undersigned were appointed a Committee to secure competent legal advice and report upon the application for membership of Dr. J. Q. Harris, of this city.

In compliance with the directions contained in the resolution under which they were appointed. Your Committee have given the subject careful consideration and respectfully present the following conclusions.

1st. That the Diploma of Dr. Harris is regular and valid, and under the act to incorporate Medical Societies for the purpose of regulating the practice of Physic and Surgery in this State, is sufficient to empower the said Harris to practice Physic or Surgery or both as set forth in his Diploma in any part of the State.

2d. That in the absence of any known declaration on the part of Dr. Harris in violation of spirit or letter of the By-Laws of the Society, the said Harris is entitled to the rights and privileges of membership in this Society.

In these conclusions, the Committee are supported by the legal opinion herewith presented of Messrs. Bowen, Rogers & Locke, Attorneys of this city.

All of which is respectfully submitted,

THOS. LOTHROP,<br>E. R. BARNES,<br>C. C. WYCKOFF, } Committee.

OFFICE OF BOWEN, ROGERS & LOCKE,<br>COUNSELLERS AT LAW.<br>BUFFALO, JANUARY 13, 1873.

DRS. LOTHROP, BARNES & WYCKOFF:

GENTLEMEN:—You ask us to answer the following question:

1st. Does the presentation of a Certificate of Membership in the Medical Society of another county of this State, entitle the holder to admission to the Erie County Medical Society.

2d. Is the Society justified in refusing a request of admission when the applicant has been guilty of practices prohibited by the By-Laws.

By the Act of the Legislature under which Medical Societies are incorporated they are severally allowed to make such By-Laws and Regulations relative to the admission and expulsion of members as they may deem proper, provided such regulations be not inconsistent with law or the rules and regulations of the State Society.

The action of each Society as to applicants for membership is based upon its own By-Laws alone, and its rights cannot be abridged by the action of any other Society.

We understand that the rules and regulations of the State Society are silent upon this question, and that the By-Laws of the Erie County Society have not been modified since 1862, so far as membership is concerned.

If we are right in these premises we are very clear that the holder of a certificate of membership in another Society, cannot for that reason demand an admission to the Society you represent.

His diploma and certificate are simply presumptive evidence of his fitness, and like all other evidence may be insufficient to make proof.

Second.—It is the settled law of this State, that the By-Laws of a Medical Society are inoperative except as to members.

Every Physician or Surgeon may demand a share in the enjoyment of the franchise of your Society, under the By-Laws as adopted.

He must be a resident of the County, of temperate habits, good moral character, and legally authorized to practice Physic or Surgery in this State. If he possess these qualifications he is presumptively entitled to membership.

You can only avoid this presumption and its results by showing *subsisting* causes clearly demanding immediate expulsion.

For instance the Court acting in the discretion allowed it would refuse a mandamus to an applicant who admitted his practice to be inconsistent with your By-Laws, and who declared his intention to so act as to violate them in the future.

This would be subsisting cause for present expulsion, but this is the extent of the limitation.

You cannot condemn a man from his record, if at the time of his application he be otherwise eligible. Yours, Very Respectfully,

BOWEN, ROGERS & LOCKE,

Dr. Storck presented resolutions recommending action of the Society to obtain a Law to protect the public from those who assume the name and functions of Physicians, but have no credentials, are not members of any recognized Medical Society or graduates of any Medical School, who are not physicians in any sense.

The resolutions were passed, and the following committee chosen to act upon its suggestions: J. F. Miner, E. Storck, Jabez Allen, James P. White, C. C. Wyckoff.

Dr. Harrington was appointed orator. Dr. Lapp, substitute.

Dr. Gould presented a bone ejected from a patient after having remained in the bronchial tube nineteen months. Patient suffered greatly, and was not expected to recover, was soon well. Dr. Miner spoke of similar case operated upon at General Hospital, bone removed and followed by recovery. Drs. Ring and Bartlett also related cases resembling the one reported by Dr. Gould.

### AFTERNOON SESSION.

Upon the re-assembling of the Society, the minutes of the forenoon session were read and approved, and after some discusson upon professional matters, the annual election took place, resulting in the choice of the following gentlemen:

*President*—Jabez Allen.
*Vice-President*—Thomas Lothrop.
*Secretary*—David E. Chace.
*Treasurer*—Wm. C. Phelps.
*Librarian*—J. B. Samo.
*Primaay Board*—Drs. Hopkins, Folwell and Willoughby.
*Censors*—Drs. Barnes, Storck, Briggs, Wyckoff, Sloan.
*Delegate to State Medical Society*—(To fill vacancy caused by the death of Dr. Parker), Wm Gould.

On motion the meeting then adjourned,

